# Exploring the causal relationship between inflammatory cytokines and immunoinflammatory dermatoses: a Mendelian randomization study

**DOI:** 10.3389/fmed.2024.1263714

**Published:** 2024-01-31

**Authors:** Jiaxuan Li, Yining Lu, Xuelian Zhao

**Affiliations:** ^1^Department of Plastic Surgery, The Second Hospital of Hebei Medical University, Shijiazhuang, China; ^2^Department of Orthopedic Surgery, the Third Hospital of Hebei Medical University, Shijiazhuang, China

**Keywords:** immunoinflammatory dermatoses, biomarkers, Mendelian randomization, GWAS, inflammation

## Abstract

**Objectives:**

Previous studies have shown that the onset and progression of several immunoinflammatory dermatoses are closely related to specific immune-inflammatory responses. To further assess the causal relationship between 41 inflammatory cytokines and immunoinflammatory dermatoses, we used a Mendelian randomization method.

**Methods:**

Mendelian two-sample randomization utilized inflammatory cytokines from a GWAS abstract containing 8,293 healthy participants as well as psoriasis (4,510 cases and 212,242 controls), atopic dermatitis (7,024 cases and 198,740 controls), and vitiligo (131 cases and 207,482 controls). The causal relationship between exposure and outcome was explored primarily using inverse variance weighting. In addition, multiple sensitivity analyses, including MR-Egger, weighted median, simple model, weighted model, and MR-PRESSO, were simultaneously applied to enhance the final results.

**Results:**

The results showed that in clinical practice, IL-4 and IL-1RA were suggestive indicators of atopic dermatitis risk (OR = 0.878, 95% CI = 0.78–0.99, *p* = 0.036; OR = 0.902, 95% CI = 0.82–1.00, *p* = 0.045). SCGF-b was a suggestive indicator of psoriasis risk (OR = 1.095, 95% CI = 1.01–1.18, *p* = 0.023). IL-4 is a suggestive indicator of vitiligo risk (OR = 2.948, 95% CI = 1.28–6.79, *p* = 0.011).

**Conclusion:**

Our findings suggest that circulating inflammatory cytokines may play a crucial role in the pathogenesis of chronic skin inflammation. IL-4 and IL-1RA may have inhibitory roles in the risk of developing atopic dermatitis, while SCGF-b may have a promoting role in the risk of developing psoriasis. Furthermore, IL-4 may contribute to the risk of developing vitiligo. These results provide insights into further understanding the mechanisms of chronic skin inflammation and offer new targets and strategies for the prevention and treatment of related diseases.

## Introduction

Immunoinflammatory dermatoses, including psoriasis, atopic dermatitis, and vitiligo, are prevalent clinical skin disorders characterized by immune dysfunction and the infiltration of inflammatory cells in the affected skin areas (1–3). The development and progression of these conditions are associated with aberrant activation of the immune system and the persistence of inflammatory responses. Psoriasis is a chronic inflammatory skin disease characterized by congenital and acquired immune abnormalities (4, 5), hyperproliferation, and aberrant differentiation of epidermal keratinocytes (6) and is often associated with arthritis or cardiometabolic disease (7, 8). In addition, proteomic characterization of psoriatic lesions reveals dermal fibroblast dysfunction, up-regulation of inflammatory cytokines, and signaling or down-regulation of structural molecules (9, 10). Skin inflammation in psoriasis may harbor certain intestinal bacteria, such as *Staphylococcus aureus* and *Streptococcus* daniels, which can exacerbate skin inflammation (11). Atopic dermatitis is an important chronic or recurrent inflammatory skin disease that usually precedes asthma and allergic diseases (12, 13). New insights into the genetics and pathophysiology of atopic dermatitis point to abnormalities in the structure of the epidermis, as well as immune dysregulation, as playing an important role not only in the development of this skin disease but also in asthma and allergy (14). The exact pathogenesis of vitiligo remains elusive and ample evidence exists to suggest changes in the immune process in vitiligo, especially in chronic and progressive diseases (15, 16). The immune system’s innate and adaptive immunity appear to be involved as either primary events or secondary outcomes.

Inflammatory cytokines play a crucial role in the pathogenesis of chronic inflammatory skin diseases (17). A better understanding of the inflammatory pathways involved could lead to targeted therapies. The abnormal activation of the immune system leads to the excessive accumulation of immune cells and the release of inflammatory factors. Within these diseases, specific immune cells such as T cells, B cells, and macrophages become activated and aggregate in the affected skin regions, thereby releasing a diverse array of inflammatory cytokines, including growth factors, chemokines, and interleukins.

Inflammatory cytokines also play a crucial role in the immune response. Inflammatory cytokines exert their influence on immune cell function and the development of inflammatory responses by binding to receptors on the surface of immune cells and activating intricate signaling pathways. Research has demonstrated that TNF-α plays a role in the formation of inflammatory skin lesions, stimulating the production of inflammatory mediators and increasing vascular permeability (18). L-17 promotes the proliferation of keratinocytes and the infiltration of inflammatory cells (19). IL-23 is closely associated with IL-17 and enhances IL-17 production through the activation of T-cells and immune cells (19, 20). IL-31 is implicated in the development of skin itching (21). These inflammatory cytokines, along with the pathways they engage, play a significant role in the development of inflammatory skin diseases. Furthermore, the immune-inflammatory response involves other molecules and pathways. For instance, inflammatory mediators and cytokines can activate the nuclear transcription factor NF-κB, which exhibits increased activity in chronic skin inflammation, resulting in heightened expression of inflammatory genes (22). Additionally, recent studies have indicated the involvement of immune cells such as T cells and dendritic cells in the initiation and progression of chronic skin inflammation (23, 24).

In this study, for the first time, we extracted validated genetic variants from published genome-wide association study (GWAS) pooled data for 41 inflammatory cytokines to investigate their association with three autoimmune dermatoses. Mendelian random (MR) analysis methods utilize genetic variation in non-experimental data to infer causal effects of exposure on outcomes. Because alleles are randomly assigned during meiosis, MR reduces traditional confounding variables and reverse causation, thus providing better evidence for causal inference (25). Two-sample MR analyses allow researchers to assess associations between instrument exposure and instrument outcome in two independent population samples, thereby improving the applicability and validity of the test.

## Method

### Mendelian randomization

Mendelian randomization is an analytical method used to assess causal relationships between observed modifiable exposures or risk factors and clinically relevant outcomes. Genome-wide association studies (GWAS) have identified tens of thousands of common genetic variants that are associated with hundreds of complex traits (26). This provides a valuable tool for studying causality, especially when randomized controlled trials are not feasible or when observational studies are subject to confounding or reverse causation leading to association bias. To address these issues, Mendelian randomization uses genetic variants as instrumental variables for testing exposure. These exposure-associated alleles of genetic variants are randomly assigned and are not subject to reverse causation. Due to the wide availability of published genetic associations, screening for appropriate genetic instrumental variables makes Mendelian randomization a time-and cost-effective method and is becoming increasingly popular for assessing and screening for potential causal associations. The observed associations between genetic instrumental variables and outcomes support the hypothesis that there is a causal relationship between the exposures and outcomes discussed. This approach helps to overcome the difficulty of conducting randomized controlled trials while mitigating association bias in observational studies due to confounding or reverse causation. Thus, Mendelian randomization provides a powerful tool for studying complex traits and potential causal relationships (27).

### Data resources

**Figure 1:**
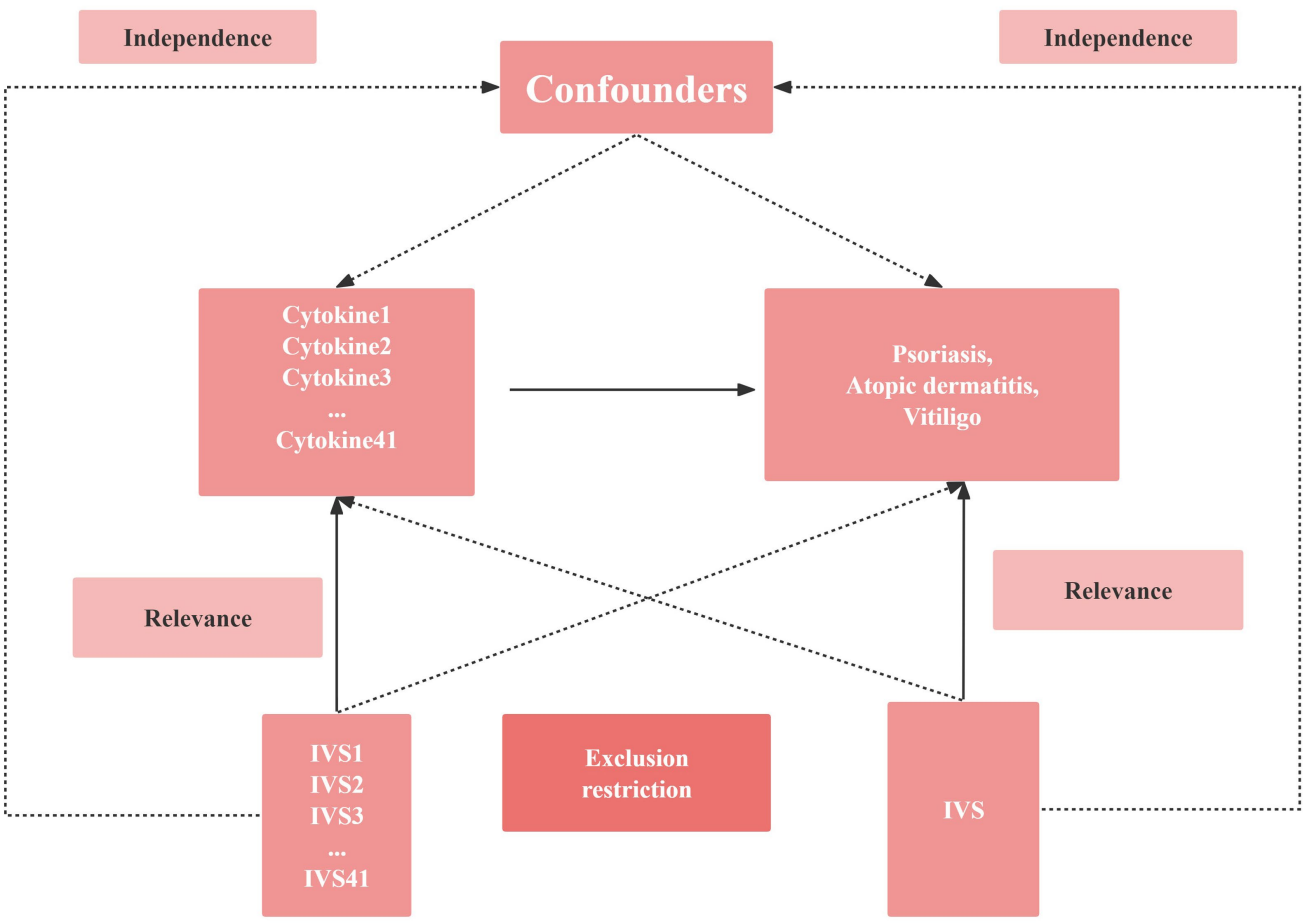
Schematic diagram of the study design in this Mendelian randomization (MR) analysis. Forty-one important instrumental variables for inflammatory cytokines and Chronic inflammatory skin diseases were selected and then explored for bidirectional causality. The three basic assumptions of MR analysis, namely correlation, independence, and exclusionary restrictions, are illustrated in this causally directed acyclic graph. IVS, instrumental variables.

The study design included atopic dermatitis cases from a meta-analysis study that included 7,024 cases and 198,740 controls of European ancestry,^1^ psoriasis cases from a meta-analysis study that included 4,510 cases and 212,242 controls of European ancestry^2^, and vitiligo cases from a meta-analysis study that included 131 cases and 207,482 controls of European ancestry (Figure 1).^3^ For the genetic instrument of cytokines, summary statistics were taken from the most comprehensive and extensive cytokine GWAS; the GWAS cytokine meta-analysis included 8,293 Finns from three independent population cohorts: the Young Finns Cardiovascular Risk Study, the FINRISK 1997 and the FINRISK 2002 studies (28). The survey was conducted in Finland, with participants aged 25 to 74 years randomly selected from five different geographic regions. Cytokine levels were measured in the participants’ EDTA plasma, heparin plasma and serum. Only measurements within the detectable range of each cytokine were included in the analysis, and any cytokines missing more than 90% of their values (48 of 7) were excluded. All participants provided written informed consent.

### Selection of cytokine SNPs

MR analysis has three core assumptions, namely correlation, independence, and exclusion restriction (29). It is assumed that the selected genetic variants are associated with risk factors (correlation) but not with any confounders in the risk factor-outcome association (independence) and that they are not associated with the outcome through any pathway other than the risk factor of interest (exclusion restriction). In this two-way study, four GWAS, 41 inflammatory cytokines and three autoimmune dermatoses were utilized. First, we used *p* < 5 × 10^−8^ as a genome-wide significance threshold to select SNPs strongly associated with three autoimmune dermatoses and inflammatory cytokines. Second, to avoid linkage disequilibrium, we clustered these SNPs (kb = 10,000, *r*^2^ = 0.001). Palindromic SNPs were discarded because we could not identify these SNPs in exposure and outcome of GWASs in systemic inflammatory regulators were aligned in the same direction. Third, the R^2^ value of each SNP was used to calculate the proportion of variance in exposure, and the F statistic was used to estimate the instrumental strength to avoid weak instrumental bias (30, 31). Finally, we will replace the unavailable SNPs in the result summary with the proxy SNPs (*R*^2^ > 0.8) from LDlink.^4^

### Statistical analysis

Since each cytokine has a different number of SNPs, we chose the Wald ratio as the primary MR analysis in cytokines with only one SNP. We chose inverse variance weighted (IVW) as the primary MR analysis in people with two or more SNPs, to assess the potential pathogenic role of inflammatory cytokines and the risk of autoimmune dermatoses. Subsequently, we performed a Cochrane Q test on IVW to detect heterogeneity. No heterogeneity was observed for most outcomes, with *p* values greater than 0.05. Only a few showed heterogeneity, but our primary MR analysis was IVW; heterogeneity can be present in it, so the presence of heterogeneity in individual outcomes does not have much impact on the prediction of causality.

Next, to further assess causality and investigate the presence of pleiotropy, we performed a set of checks, including MR Egger regression and MR-PRESSO. In addition, Leave-one-out was used to analyze the possibility of individual SNPs confounding the overall MR analysis. We also used PhenoScanner to examine potential dimorphic phenotypes in the evaluated individual SNPs to eliminate their potential influence on the results. Most of the above work was performed in R analysis software (version 4.0.3) for the relevant R packages, including two sample MRs, data arrays, etc.

## Result

Details of the study and dataset are in Supplementary Table S1, the participants were all European (100%), overcoming ethnic differences.

### Causal relationship between IL-4 and IL-1RA and atopic dermatitis

**Figure 2:**
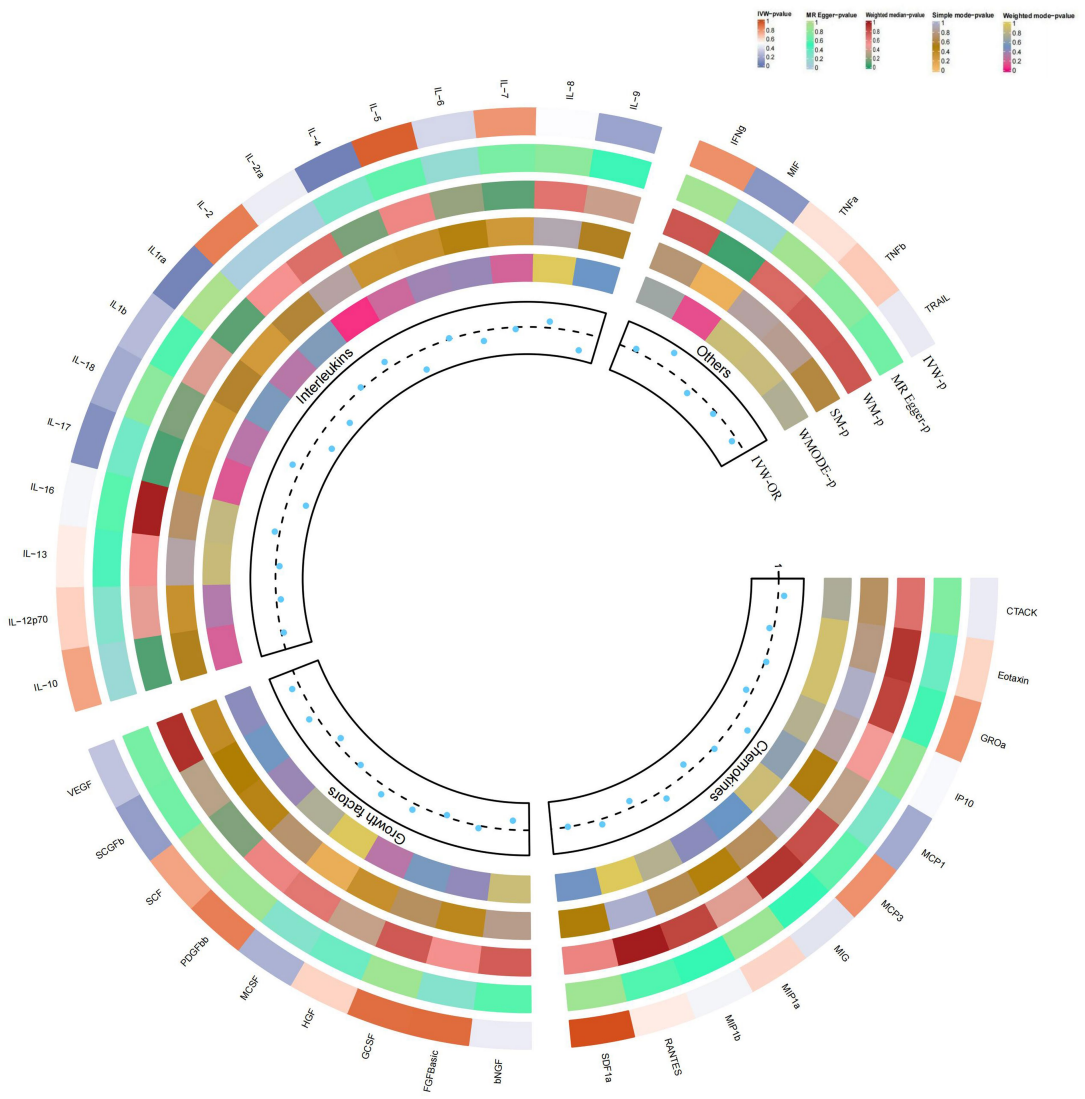
Causal correlations of 41 inflammatory cytokines on atopic dermatitis. The change in the odds ratio (OR) of atopic dermatitis per one-SD rise in the cytokine level is shown by OR and 95% confidence interval. *p*-value 0.05/41 = 0.0012 was found significant after multiple-comparison correction. The results from the inverse variance weighted method were shown for all cytokines. bNGF, beta nerve growth factor; CTACK, cutaneous T cell-attracting chemokine; FGFBasic, basic fibroblast growth factor; GCSF, granulocyte colony-stimulating factor; GROa, growth-regulated oncogene-a; HGF, hepatocyte growth factor; IFNg, interferon gamma; IL, interleukin; IP, interferon gamma-induced protein 10; MCP1, monocyte chemotactic protein 1; MCP3, monocyte-specific chemokine 3; MCSF, macrophage colony-stimulating factor; MIF, macrophage migration inhibitory factor; MIG, monokine induced by interferon gamma; MIP1a, macrophage inflammatory protein-1a; MIP1b, macrophage inflammatory protein-1b; PDGFbb, platelet-derived growth factor BB; RANTES, regulated upon activation normal T cell expressed and secreted factor; SCF, stem cell factor; SCGFb, stem cell growth factor beta; SDF1a, stromal cell-derived factor-1 alpha; SNPs, single-nucleotide polymorphisms; TNFa, tumor necrosis factor alpha; TNFb, tumor necrosis factor beta; TRAIL, TNF-related apoptosis-inducing ligand; VEGF, vascular endothelial growth factor*.

**Figure 3:**
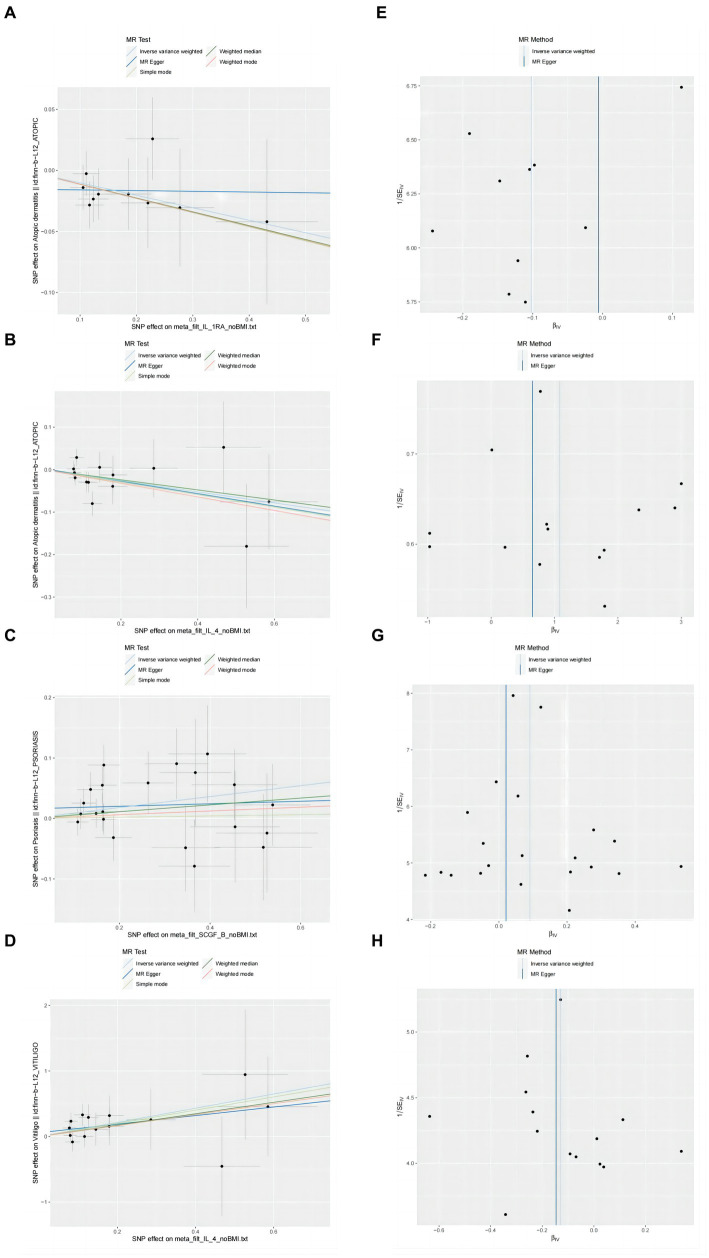
Scatter plots and funnel plots of MR analyses for IL-1RA IL-4, SCGF-b and IL-4 in psoriasis, atopic dermatitis, vitiligo. **(A–D)** Individual inverse variance (IV) associations with cytokine risk are displayed versus individual IV associations with Chronic inflammatory skin diseases in black dots. The 95%CI of odd ratio for each IV is shown by vertical and horizontal lines. The slope of the lines represents the estimated causal effect of the MR methods. **(E–H)** The funnel plots show the inverse variance weighted MR estimate of each cytokine single-nucleotide polymorphism with Chronic inflammatory skin diseases versus 1/standard error (1/SEIV).

Our findings suggest a potential role for IL-4 and IL-1RA in the risk of developing atopic dermatitis according to the IVW method (Figure 2). By using the IVW method, we found that higher levels of IL-4 and IL-1RA genetic prediction were associated with a lower risk of atopic dermatitis, OR = 0.878, 95% CI = 0.78–0.99, *p* = 0.036 per 1 standard deviation (SD); OR = 0.902, 95% CI = 0.82–1.00, *p* = 0.045 per 1 standard deviation (SD) (Figures 3A,B,E,F). Using Cochran’s Q test, we also did not observe heterogeneity (*p* = 0.478; *p* = 0.939), nor did we find directional polymorphism (MR egger-intercept = 0.002, *p* = 0.892 for MR egger-intercept; *p* = 0.48 for MR PRESSO global test; MR egger-intercept = −0.015, P for MR egger-intercept = 0.489; P for MR PRESSO global test = 0.947). Except for IL-4 and IL-1RA, none of the other cytokines (e.g., VEGF, GRO-α, Trail, MIG, IL-7, IL-17) were shown to be associated with the risk of osteonecrosis in the IVW primary MR analysis or other secondary analyses. In the heterogeneity test, we found significant heterogeneity for SCF (*p* = 0.001), MIG (*p* = 0.002), FGFBasic (*p* = 0.008), IL-5 (*p* = 0.021), MIP1b (*p* = 0.027), and GCSF (*p* = 0.037), whereas the majority of the other cytokines demonstrated significant non-heterogeneity. Our MR-egger regression did not find any polymorphism in the *p*-values of all cytokines except IL-2 (*p* = 0.022 for MR egger-intercept). Finally, our MR-PRESSO assay as an additional robustness test did not find any outliers except for MIG (*p* = 0.01), FGFBasic (*p* = 0.016), IL-5 (*p* = 0.024) and MIP1b (*p* = 0.037; Supplementary Tables S1–S3).

### Causal relationship between SCGF-b and psoriasis

**Figure 4:**
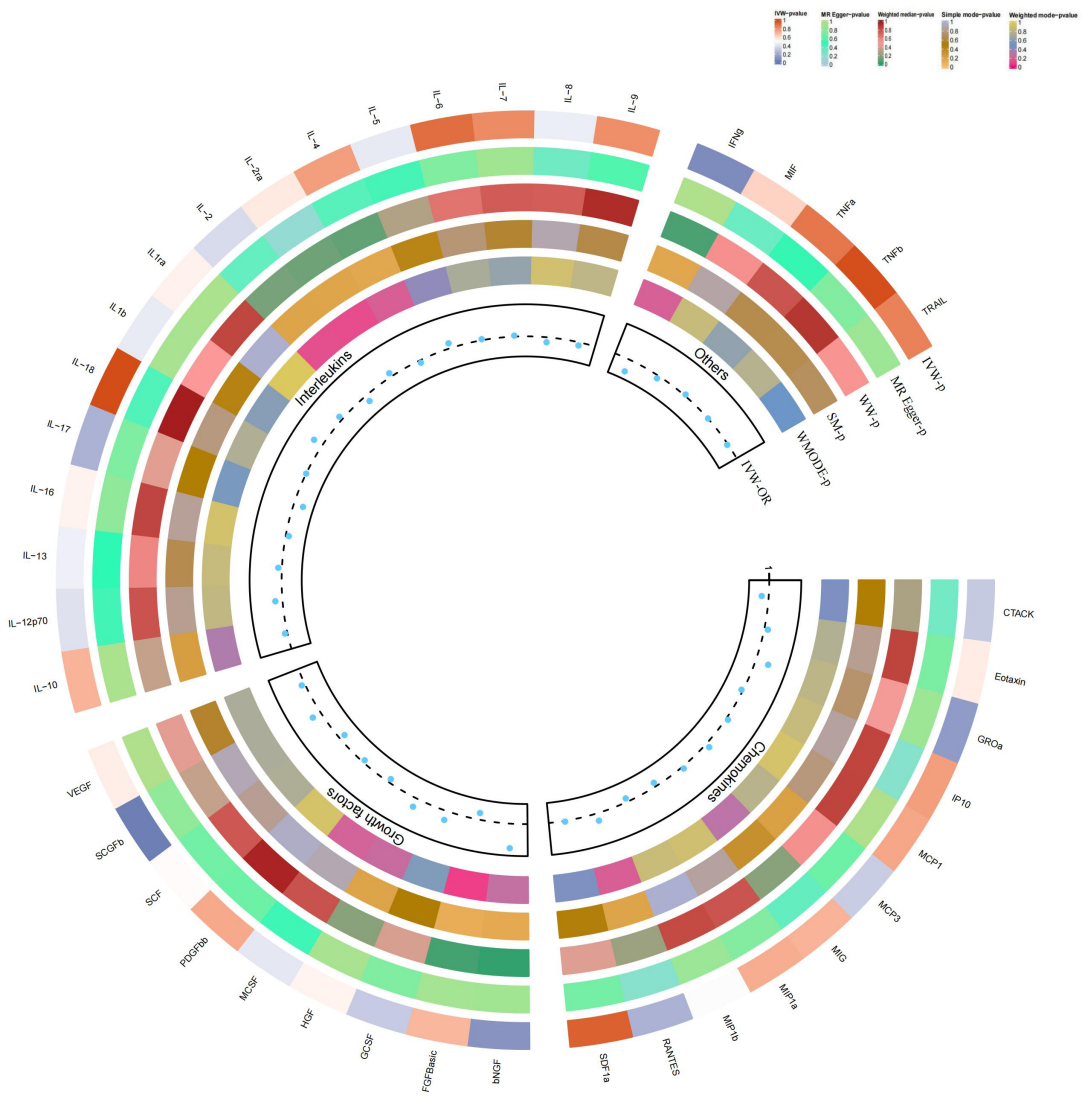
Causal correlations of 41 inflammatory cytokines on psoriasis. The change in the odds ratio (OR) of psoriasis per one-SD rise in the cytokine level is shown by OR and 95% confidence interval. *p*-value 0.05/41 = 0.0012 was found significant after multiple-comparison correction. The results from the inverse variance weighted method were shown for all cytokines.

In the present study, based on the IVW approach, we identified a potential role of SCGF-b in the risk of psoriasis development (Figure 4). By using the IVW method, we found that higher levels of SCGF-b genetic prediction were associated with a higher risk of psoriasis, (OR = 1.095, 95% CI = 1.01–1.18, *p* = 0.023 per 1 standard deviation (SD); Figures 3C,G). Using Cochran’s Q test, we also did not observe heterogeneity (*p* = 0.478), nor did we find directional polymorphism (MR egger-intercept = 0.015, *p* = 0.349 for MR egger-intercept; *p* = 0.538 for MR PRESSO global test). Except for SCGF-b, none of the other cytokines (e.g., VEGF, GRO-α, Trail, MIG, IL-7, IL-17) were shown to be associated with the risk of osteonecrosis in the IVW primary MR analysis or other secondary analyses. In the heterogeneity test, we found significant heterogeneity for GROa (*p* = 0.001), FGFBasic (*p* = 0.007), IL-9 (*p* = 0.016), and HGF (*p* = 0.021), whereas most of the other cytokines showed significant non-heterogeneity. Furthermore, our MR-egger regression did not reveal any polymorphism in the *p*-values of all cytokines. Except for GROa (*p* = 0.003), our MR-PRESSO assay as an additional robustness test did not find any outliers (Supplementary Tables S4–S6).

### Causal relationship between IL-4 and vitiligo

**Figure 5:**
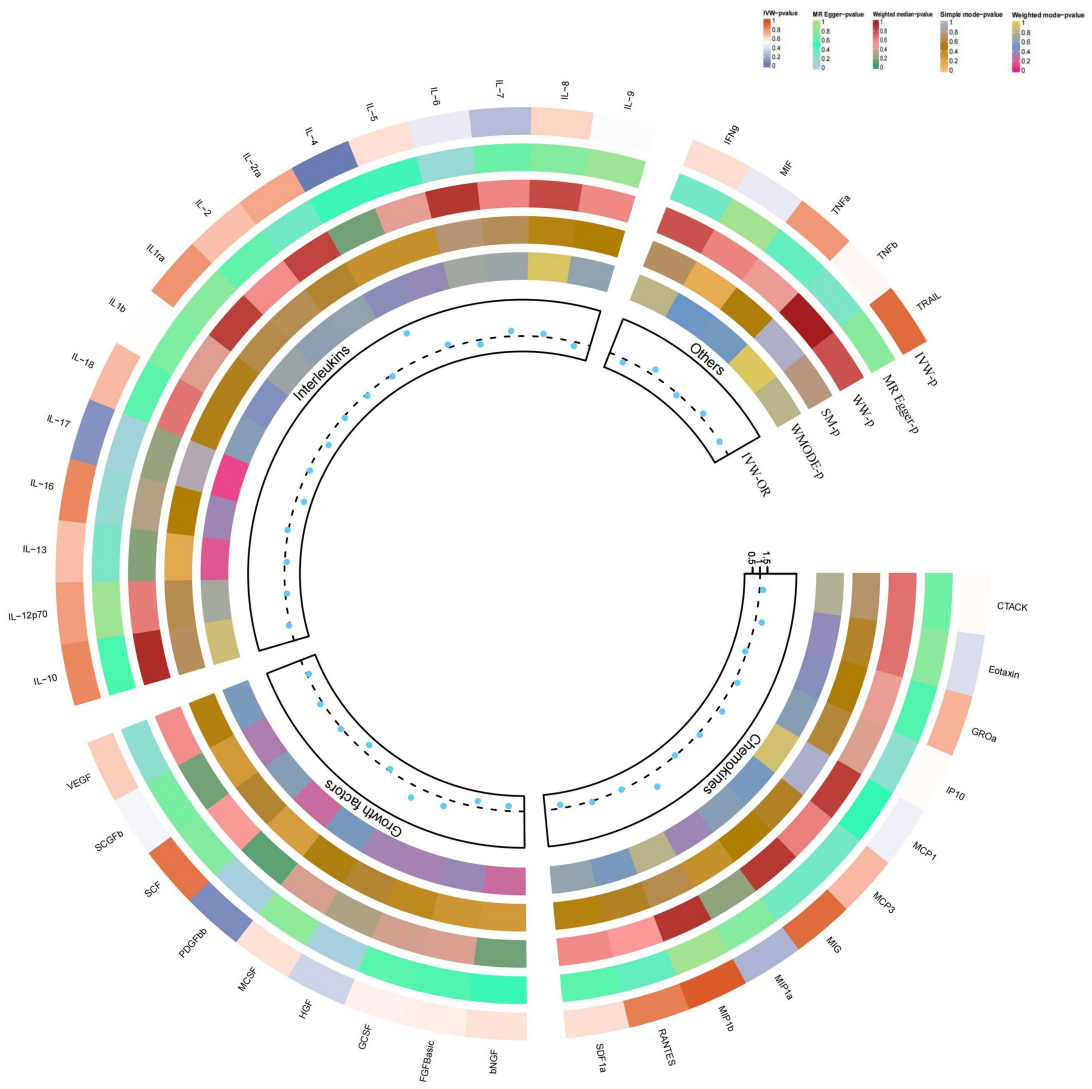
Causal correlations of 41 inflammatory cytokines on vitiligo. The change in the odds ratio (OR) of vitiligo per one-SD rise in the cytokine level is shown by OR and 95% confidence interval. *p*-value 0.05/41 = 0.0012 was found significant after multiple-comparison correction. The results from the inverse variance weighted method were shown for all cytokines.

By using the IVW method, we found that higher levels of IL-4 genetic prediction were associated with a higher risk of vitiligo, (OR = 2.948, 95% CI = 1.28–6.79, *p* = 0.011 per 1 standard deviation (SD); Figure 5; Figures 3D,H). Using Cochran’s Q test, we also did not observe heterogeneity (*p* = 0.836), nor did we find directional polymorphism (MR egger-intercept = 0.059, *p* = 0.592 for MR egger-intercept; *p* = 0.842 for MR PRESSO global test). Except for IL-4, none of the other cytokines (e.g., VEGF, GRO-α, Trail, MIG, IL-7, IL-17) were shown to be associated with the risk of osteonecrosis in the IVW primary MR analysis or other secondary analyses. In the heterogeneity test, we found significant heterogeneity for IL-13 (*p* = 0.037), while most of the other cytokines showed significant non-heterogeneity. In addition, our MR-egger regression did not find any polymorphism in the *p*-values of all cytokines. Finally, our MR-PRESSO assay as an additional robustness test did not reveal any outliers except for IL-13 (*p* = 0.044) (Supplementary Tables S7–S9).

## Discussion

Inflammatory factors play a pivotal role in the development of autoimmune dermatoses. Abnormal activation of the immune system triggers the accumulation of immune cells and the release of inflammatory factors. The release of these inflammatory mediators initiates a persistent inflammatory response, leading to abnormal changes in the skin tissue, including abnormal keratinization, impaired epidermal barrier function, and vasodilation (17, 23). This vicious cycle further stimulates the release of immune cells and inflammatory factors, exacerbating the persistence of chronic inflammation. In this study, we employed MR analysis methods to investigate the potential role of circulating cytokines in the risk of developing these diseases, focusing specifically on the causal relationship between circulating cytokines and atopic dermatitis, psoriasis, and vitiligo.

Several studies have now revealed the potential role of IL-1RA in the etiology and treatment of acne (32, 33). Our study provides new proof of the above ideas in a genetic perspective. Using the IVW method, we observed that higher genetic prediction levels of IL-1RA were associated with a lower risk of atopic dermatitis. The mechanisms and pathways involved in the risk reduction of IL-1RA in atopic dermatitis are complex. IL-1RA is an antagonist of IL-1 and inhibits IL-1 activity by competitively binding to the IL-1 receptor (34). Interleukin (IL)-1 family cytokines initiate inflammatory responses and modulate innate and adaptive immunity. While they play a crucial role in host defense, excessive immune activation can also lead to the development of chronic inflammatory diseases (35). By inhibiting the signaling pathway of IL-1, IL-1RA reduces the release of inflammatory mediators and the activation of inflammatory responses. Studies have shown that IL-1RA reduces inflammatory responses and skin damage by inhibiting the activity of IL-1 (36, 37). These cytokines interact with each other through various mechanisms, jointly regulating the pathogenesis of atopic dermatitis. The combined findings suggest that the reduced risk of atopic dermatitis associated with IL-1RA may be realized through multiple pathways, including immune regulation, inflammatory modulation, and restoration of skin barrier function.

SCGFb (Stem Cell Growth Factor Beta) is a newly discovered secreted sulfated glycoprotein that functions as a growth factor in early hematopoiesis (38). It is selectively produced by bone and hematopoietic stromal cells and can mediate its proliferative activity on primitive hematopoietic progenitor cells (39). Several studies have shown that SCGF-b is causally associated with a variety of immune-related diseases, but the subsequent mechanisms have not been revealed (40–42). Nevertheless, this novel biomarker may also complement the limitations of conventional biomarkers in routine clinical practice.

Psoriasis is an immune-mediated chronic skin disease characterized by the aberrant activation of multiple immune cells and inflammatory factors (43, 44). Currently, no studies have revealed a potential link between SCGF-b and psoriasis. However, our study found that SCGF-b may be associated with an increased risk of psoriasis development, with higher genetic prediction levels of SCGF-b being associated with a higher risk. Numerous studies have shown that abnormal proliferation and differentiation of stem cells are closely related to the development of psoriasis and are involved in the immunomodulatory process of the disease (45–47). Furthermore, SCGF-b has been associated with the prognosis of Crohn’s disease (CD), and considering the potential causal relationship between CD and psoriasis, SCGF-b may also have a role in psoriasis (48, 49). A comprehensive understanding of the function and regulatory mechanisms of SCGF-b can help uncover the pathophysiological mechanisms of psoriasis and provide new targets and strategies for its treatment and intervention.

Finally, we found that IL-4 had important associations with the risk of immunoinflammatory dermatoses. First, higher genetically predicted levels of IL-4 were associated with a higher risk of vitiligo. This suggests that IL-4 may contribute to the pathogenesis of vitiligo. IL-4 is involved in immunomodulation and affects melanocyte function and proliferation by regulating the JAK2-STAT6 pathway (50). Additionally, IL-4 promotes the inflammatory response by stimulating the proliferation and activation of inflammatory cells, leading to an inflammatory response (51). The infiltration of inflammatory cells and the release of inflammatory mediators can cause damage to surrounding melanocytes, exacerbating the onset of pigmentary disorders (52, 53). Elevated levels of IL-4 may affect the activation status of immune cells, leading to increased attack on melanocytes and further promoting the development of vitiligo (54).

Some IL-4 findings in vitiligo studies seem to be contrary to ours (55, 56). We believe that there may be differences in experimental design and methodology between the previous studies and ours. This includes aspects such as sample selection, analytical techniques, and experimental conditions. In addition, biological differences between different patient populations may influence the expression of IL-4 levels. Previous studies and our study may involve different patient groups, which may be one of the reasons for the conflicting results. Vitiligo may undergo different inflammatory stages, and IL-4 expression levels may vary between these stages. Previous studies and our study may have targeted different stages of the vitiligo disease process, leading to inconsistent results. A comprehensive understanding of the function and regulatory mechanisms of IL-4 can help unravel the pathophysiological mechanisms of vitiligo and provide new targets and strategies for its treatment and intervention.

Furthermore, while it is noteworthy that higher IL-4 levels in our study were associated with a lower risk of atopic dermatitis, several studies have demonstrated the involvement of IL-4 in the onset and development of atopic dermatitis (57, 58). Our study may reveal additional pathways through which IL-4 is involved in atopic dermatitis. IL-4, a cytokine secreted by Th2 cells, regulates immune responses and inflammatory processes by activating its receptors (57). It inhibits the Th1 immune response, reduces the infiltration of inflammatory cells and inflammatory responses, and promotes the production of the anti-inflammatory cytokine IL-10 (59). Additionally, IL-4 and IL-1RA act by regulating inflammation-related molecules and pathways. IL-4 inhibits the production of multiple inflammatory mediators, such as IL-17, IL-22, and TNF-α, while promoting skin barrier repair and protection.

It is necessary to point out some limitations of this study. First, our study used MR analysis to infer causality, but was unable to consider potential confounding factors. For example, we could not know whether the patients in this study had other diseases that could cause inflammation. Therefore, our results need to be validated in larger studies. Second, we only considered the role of a few cytokines, while other unexplored cytokines may also play a key role in chronic skin inflammation. Third, MR analysis can only be used to infer causality and cannot yet elaborate the causal relationship between the study population and disease severity or duration, and further studies could investigate a broader network of cytokines and their interactions. Fourth, individual differences, different disease stages, and patient treatment history may all contribute to fluctuations in cytokine (IL-4) levels. There may be complex networks of interactions with other cytokines that may also have an impact on the results. Fifth, instrumental variables from different analytical platforms, experiments, populations, etc. may be heterogeneous, thus affecting the results of Mendelian randomization analysis. Finally, we must point out that the two-sample Mendelian randomization method also has some limitations. In two-sample Mendelian randomization, the dataset used to perform the Mendelian randomization is the same as the dataset used to identify the instruments. This can lead to the phenomenon of the winner’s curse, i.e., GWAS data can lead to an overestimation of genetic effect sizes (60, 61). If the instruments in GWAS are not sufficiently accurate, then the results of MR may be biased, thus affecting the accuracy of causality. In addition, since we were unable to provide the original dataset prior to harmonization, this could lead to bias due to improper data harmonization (62).

In summary, our findings suggest that circulating inflammatory cytokines may play a crucial role in the pathogenesis of chronic skin inflammation. IL-4 and IL-1RA may have inhibitory roles in the risk of developing atopic dermatitis, while SCGF-b may have a promoting role in the risk of developing psoriasis. Furthermore, IL-4 may contribute to the risk of developing vitiligo. Studies have shown that changes in cytokine levels caused by targeted therapies alter disease symptoms (63, 64). Our results provide insight into further understanding the mechanisms of chronic skin inflammation and offer new targets and strategies for the prevention and treatment of immunoinflammatory dermatoses.

## Data availability statement

The original contributions presented in the study are included in the article/Supplementary material, further inquiries can be directed to the corresponding author.

## Author contributions

JL: Data curation, Software, Writing – original draft. YL: Conceptualization, Writing – original draft. XZ: Conceptualization, Writing – review & editing.

## Footnotes

^1^https://gwas.mrcieu.ac.uk/datasets/finn-b-L12_ATOPIC/)

^2^https://gwas.mrcieu.ac.uk/datasets/finn-b-L12_PSORIASIS/)

^3^https://gwas.mrcieu.ac.uk/datasets/finn-b-L12_VITILIGO/)

^4^https://ldlink.nih.gov/?tab=home)
